# Role of voltage gated Ca^2+^ channels in rat visceral hypersensitivity change induced by 2,4,6-trinitrobenzene sulfonic acid

**DOI:** 10.1186/1744-8069-9-15

**Published:** 2013-03-28

**Authors:** Aihua Qian, Dandan Song, Yong Li, Xinqiu Liu, Dong Tang, Weiyan Yao, Yaozong Yuan

**Affiliations:** 1 Department of Gastroenterology, Ruijin Hospital, Shanghai Jiaotong University School of Medicine, Shanghai 200025, China; 2 Department of Biochemistry and Molecular Cell Biology, Shanghai Jiaotong University School of Medicine, Shanghai, China; 3 Department of Neurobiology, Shanghai Jiaotong University School of Medicine, Shanghai, China

**Keywords:** Dorsal root ganglia, TNBS, Visceral sensory, Voltage gated calcium channels, [Ca^2+^]_i_

## Abstract

**Background:**

Visceral pain is common symptom involved in many gastrointestinal disorders such as inflammatory bowel disease. The underlying molecular mechanisms remain elusive. We investigated the molecular mechanisms and the role for voltage gated calcium channel (VGCC) in the pathogenesis in a rat model of 2,4,6-trinitrobenzenesulfonic acid (TNBS) induced visceral inflammatory hypersensitivity.

**Results:**

Using Agilent cDNA arrays, we found 172 genes changed significantly in dorsal root ganglia (DRG) of TNBS treated rats. Among these changed genes, Cav1.2 and Cav2.3 were significantly up-regulated. Then the RT-PCR and Western blot further confirmed the up-regulation of Cav1.2 and Cav2.3. The whole cell patch clamp recording of acutely dissociated colonic specific DRG neurons showed that the peak *I*_Ba_ density was significantly increased in colonic neurons of TNBS treated rats compared with control rats (−127.82 ± 20.82 pA/pF Vs −91.67 ± 19.02 pA/pF, n = 9, *P < 0.05). To distinguish the different type of calcium currents with the corresponding selective channel blockers, we found that L-type (−38.56 ± 3.97 pA/pF Vs −25.75 ± 3.35 pA/pF, n = 9, * P < 0.05) and R-type (−13.31 ± 1.36 pA/pF Vs −8.60 ± 1.25 pA/pF, n = 9, * P < 0.05) calcium current density were significantly increased in colonic DRG neurons of TNBS treated rats compared with control rats. In addition, pharmacological blockade with L-type antagonist (nimodipine) and R-type antagonist (SNX-482) with intrathecal injection attenuates visceral pain in TNBS induced inflammatory visceral hypersensitivity.

**Conclusion:**

Cav1.2 and Cav2.3 in colonic primary sensory neurons play an important role in visceral inflammatory hyperalgesia, which maybe the potential therapeutic targets.

## Background

Abdominal pain and discomfort are amongst the most vexing symptoms for patients suffering from inflammatory bowel diseases such as Crohn’s ileitis and present a major therapeutic challenge for physicians. The underline mechanisms are still unknown. Therefore, it is essential to identify the detailed molecular mechanisms that are involved in inflammatory/infectious events leading to visceral hypersensitivity both for understanding underlying mechanisms and developing new therapies.

In the visceral sensory neurotransmission process, the neurons that relay sensory information from the intestine to the spinal cord are the dorsal root ganglia (DRG) neurons. These pseudounipolar neurons have a peripheral axon that travels to the intestine and a central axon that project to sensory neurons in the dorsal horn of the spinal cord. Taken together, current knowledge suggests that changes in gene expression in DRGs may contribute to the generation and development of inflammatory visceral hypersensitivity. Proposed mechanisms that could account for the sensitization of primary sensory afferents include increased expression of receptor molecules involved in nociceptive pathways or alteration of ionic channel properties. Here, we took a broader approach, a cDNA array, to gain a comprehensive overview of the alteration of gene expression in DRGs after TNBS induced visceral inflammation.

Voltage-gated calcium channels (VGCC) regulate rapid and transient changes in intracellular calcium within excitable cells. Calcium entering the cell through these channels serves as a second messenger, initiating intracellular events such as contraction, secretion, protein phosphorylation, gene expression, neurotransmitter release and action potential firing patterns [1]. Within neurons of the DRG the activity of calcium channels has been shown to play an important role in nociceptive processing [2,3] and regulation of calcium channel activity has been shown to modulate pain in clinical and experimental settings [2,4,5]. More recently, agents which modulate the activity of the calcium channel α2δ subunit have also been shown to exhibit experimentally and clinically important pain relief [6]. However, a link between changes in voltage-gated calcium channel of DRG and the occurrence of postinflammatory hypersensitivity symptoms still has to be established. The mechanism, for example, whether a specific subtype involves, or how or where they contribute to the postinflammatory visceral pain, has remained to be fully clarified, and the subtype channels could be viewed as potential therapeutic targets for the treatment of postinflammatory hypersensitivity symptoms.

Thus the aim of this study was to identify marked changes in the expression of genes in DRG neurons innervating the inflamed intestine. Then among these diverse changed molecular, we are try to developing successful therapies to target ion channels that promote the sensitization of primary sensory afferents.

## Results

### Identification of differentially expressed genes

Using cDNA microarray, we found 172 genes changed significantly in TNBS treated rats including ion channels, receptors and signal transduction-related molecules (Table 1). There were 13 channels strongly regulated in DRG of TNBS-treated rats, including Ca^2+^ channels (α1E and α1C), K^+^ channels, purinergic receptor P2X, ryanodine receptor 1(Ryr1), glutamate receptor(AMPA2), transient receptor potential cation channel(TRPC3) channels and so on (Table 1). The expression of Ca^2+^ channels α1E and α1C subunit were the most strongly up-regulated ion channels, ≈15-fold increase in the DRG 4 days after TNBS treated. At the same time, 15 receptors were founded to be strongly regulated in the DRG of TNBS-treated rats (Table 1). Among these changed receptors, we found that many receptors of inflammatory mediators such as 5-hydroxytryptamine receptor (5-HTR, 1A and 2A), prostaglandin E receptor (EP2), fibroblast growth factor receptor (FGFR) and neuropeptide Y receptor were up-regulated. In addition, a total of 22 signal transduction modulators and effectors in the DRG were significantly regulated (Table 1). Approximated 73% of these signal transduction molecules were up-regulated, indicating that the activity of these signaling pathways may generally be enhanced in the DRG after TNBS treated. Ras-related GTP binding B was the most strongly up-regulated signal transduction molecule, 12-fold increase as compared with the control. In the same time, the RAS p21 protein activator and RAS oncogene family (RAB39, RAB5A and RAB15) were also significantly up-regulated 4 days after TNBS treated. Mitogen-activated protein kinase 6 (ERK 3) and 8 (JNK 1) were also significantly up-regulated 4 days after TNBS treated.

**Table 1:** List of the strongly regulated genes in DRGs of TNBS treated rats

**Ref. or Acc. number**	**Symbol**	**Gene name**	**TNBS/Con(mean ± SD)**
**Channels**			
AY323810	Cacna1c	calcium channel, voltage-dependent, L type, alpha 1C subunit	18.45 ± 1.62
NM_019294	Cacna1e	calcium channel, voltage-dependent, L type, alpha 1E subunit	14.82 ± 1.51
XM_341818	Ryr1	ryanodine receptor 1, skeletal muscle	13.50 ± 1.23
NM_012825	Aqp4	aquaporin 4	10.26 ± 1.21
NM_019256	P2rx7	purinergic receptor P2X, ligand-gated ion channel, 7	3.75 ± 0.81
XM_233477	Kcnq4	potassium voltage-gated channel, subfamily Q, member 4	3.03 ± 0.57
XM_344426	Kctd9	potassium channel tetramerisation domain containing 9	2.76 ± 0.32
NM_017261	Gria2	glutamate receptor, ionotropic, AMPA2	2.47 ± 0.29
NM_017303	Kcnab1	Potassium voltage-gated channel, shaker-related subfamily, beta member 1	2.25 ± 0.27
NM_031046	Itpr2	inositol 1,4,5-triphosphate receptor 2	2.17 ± 0.25
AI407979	Fxyd5	FXYD domain-containing ion transport regulator 5	0.46 ± 0.04
NM_053870	Kcnj4	potassium inwardly-rectifying channel, subfamily J, member 4	0.44 ± 0.05
AF313481	Trpc3	transient receptor potential cation channel, subfamily C, member 3	0.27 ± 0.04
**Receptor**			
NM_012585	Htr1a	5-hydroxytryptamine (serotonin) receptor 1A	9.87 ± 1.32
NM_012675	Tnf	tumor necrosis factor (TNF superfamily, member 2)	8.94 ± 1.21
M64867	Htr2a	5-hydroxytryptamine (serotonin) receptor 2A	5.12 ± 0.98
NM_053429	Fgfr3	fibroblast growth factor receptor 3	4.81 ± 0.84
NM_001002853	P2ry13	purinergic receptor P2Y, G-protein coupled, 13	2.92 ± 0.45
BC087035	Tgfbr1	transforming growth factor, beta receptor 1	2.68 ± 0.46
NM_001013032	Npy1r	neuropeptide Y receptor Y1	2.31 ± 0.30
NM_031569	Oprl1	opioid receptor-like 1	2.26 ± 0.23
NM_031088	Ptger2	prostaglandin E receptor 2, subtype EP2	2.26 ± 0.25
NM_053552	Tnfsf4	tumor necrosis factor (ligand) superfamily, member 4	2.15 ± 0.14
NM_012800	P2ry1	purinergic receptor P2Y, G-protein coupled 1	2.10 ± 0.10
XM_217136	Cd3g	CD3 antigen, gamma polypeptide	0.47 ± 0.05
NM_019198	Fgf17	fibroblast growth factor 17	0.42 ± 0.06
NM_138880	Ifng	interferon gamma	0.36 ± 0.04
NM_053680	Insl3	insulin-like 3	0.02 ± 0.01
**Signal transduction-related molecules**			
AW526572	RragB	Ras-related GTP binding B	12.28 ± 1.89
XM_220933	Arf4l	ADP-ribosylation factor 4-like	6.06 ± 0.64
XM_343450	Rasa2	RAS p21 protein activator 2	4.44 ± 0.41
NM_133392	Stk17b	serine/threonine kinase 17b (apoptosis-inducing)	3.13 ± 0.30
NM_031622	Mapk6	mitogen-activated protein kinase 6 (ERK 3)	3.00 ± 0.34
XM_236242	Rab39	RAB39, member RAS oncogene family	2.73 ± 0.31
NM_134346	Rap1b	RAS related protein 1b	2.69 ± 0.36
NM_031130	Nr2f1	nuclear receptor subfamily 2, group F, member 1	2.59 ± 0.29
XM_342223	Prkci	protein kinase C, iota	2.46 ± 0.26
NM_022692	Rab5a	RAB5A, member RAS oncogene family	2.36 ± 0.26
XM_225020	Rasa3	RAS p21 protein activator 3	2.34 ± 0.24
XM_341399	Mapk8	mitogen-activated protein kinase 8 (JNK 1)	2.33 ± 0.28
NM_053522	Rhoq	ras homolog gene family, member Q	2.19 ± 0.18
M83679	Rab15	RAB15, member RAS onocogene family	2.17 ± 0.16
NM_021763	Arfip1	ADP-ribosylation factor interacting protein 1	2.14 ± 0.14
NM_053306	Pak2	p21 (CDKN1A)-activated kinase 2	2.07 ± 0.10
XM_232732	Map3k6	mitogen-activated protein kinase kinase kinase 6	0.49 ± 0.04
BF403410	Camk2b	Calcium/calmodulin-dependent protein kinase II, beta	0.46 ± 0.04
NM_031716	Wisp1	WNT1 inducible signaling pathway protein 1	0.45 ± 0.04
NM_133568	Rasd2	RASD family, member 2	0.38 ± 0.05
XM_215821	Usp8	ubiquitin specific protease 8	0.36 ± 0.04
AA945841	Rab10	RAB10, member RAS oncogene family	0.33 ± 0.05

Data are presented as mean ± SD, n = 4.

### Real time PCR and Western blot analysis of Cav1.2 and Cav2.3 expression levels

**Figure 1:**
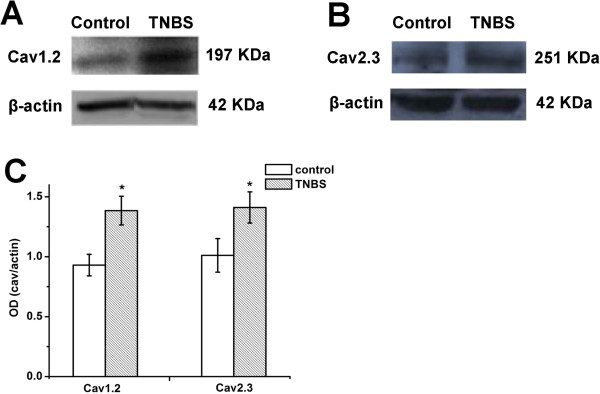
**Cav1.2 and Cav2.3 subunit protein level in L6-S2 DRGs in control and TNBS treated rats.** (**A**) Representative Western blot for Cav1.2 in extracts from L6-S2 DRGs showing a prominent band at ~197 kDa. (**B**) Representative Western blot for Cav2.3 in extracts from L6-S2 DRGs showing a prominent band at ~251 kDa. (**C**) The densitiometric analysis of western blot. Bar charts show that the expression of Cav1.2 and Cav2.3 significantly increased in TNBS treated rats (n = 5 for each group, * P < 0.05). Data are presented as mean ± SD of 5 different rats from each group.

The samples were examined by RT-PCR in terms of the above-mentioned steps to obtain cycle threshold (Ct) values. Based on the Ct values, we calculated the targeted gene expression level (ΔCt values). Table 2 shows that the mRNA expression of Cav1.2 and Cav2.3 were significantly increased in L6-S2 DRG of TNBS treated rats (n = 4 for each group, * P < 0.05). We then compared Cav1.2 and Cav2.3 protein expression levels with Western blot. We found that the protein expression level of Cav1.2 and Cav2.3 were also significantly increased in L6-S2 DRG of TNBS treated group compared with control group (Figure 1, n = 5 for each group, * P < 0.05).

**Table 2:** Real time TR-PCR analysis for quantification of Cav1.2 and Cav2.3 expression levels

**Gene bank accession no.**	**Control (ΔCt1)**	**TNBS (ΔCt2)**
AY323810	12.81 ± 0.87	9.23 ± 0.64 *
(Cav1.2)		
NM_019294	15.28 ± 1.12	12.11 ± 0.76 *
(Cav2.3)		

Data are presented as mean ± SD, n = 4, * P < 0.05.

### The voltage gated calcium current in colonic DRG neurons

**Figure 2:**
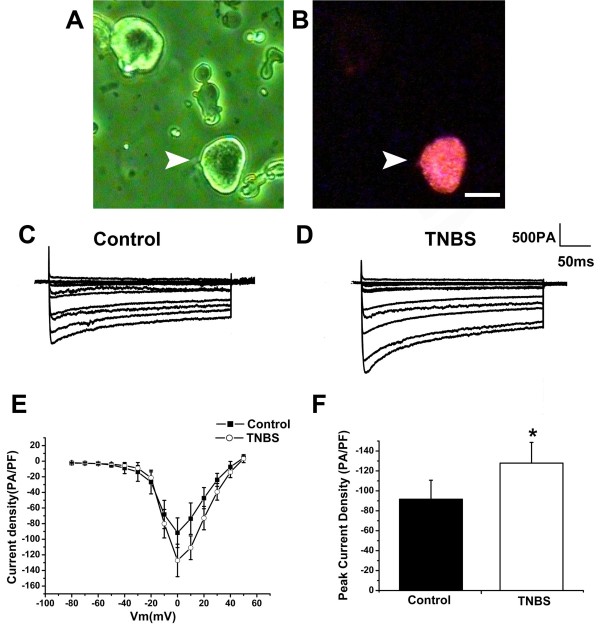
**Voltage gated Ca**^**2+ **^**current of DiI**^**+ **^**DRG neurons from control and TNBS treated rats. A**, The photomicrograph of dissociated L6-S2 DRG neurons. **B**, DiI-labeled neuron (arrow) is visualized under epifluorescence illumination. **C**, Representative total *I*Ba currents traces of DiI^+^ neurons from control rats. **D**, Representative total *I*Ba currents traces of DiI^+^ neurons from TNBS treated rats. **E**, Current–voltage relationships (I-V curves). Current–voltage plot of average data shows an increase of peak current in colonic neurons from TNBS treated rats (open circle) compared with control rats (square). **F**, Bar graph shows peak current density of *I*Ba currents. The average peak *I*Ba currents of colonic neurons from TNBS treated rats were significantly increased compared with control rats (−127.82 ± 20.82 pA/pF Vs −91.67 ± 19.02 pA/pF, n = 9 for both; * P < 0.05). Scale bar for A and B =30 *μ*m.

The colonic sensory neurons were retrograde traced with DiI and were isolated for patch clamp recording (Figure 2A and B). The total *I*_Ba_ current trace of colonic sensory neurons from control and TNBS treated rats were elicited by square-wave voltage commands (a 300 ms voltage step from −80 to 50 mV with 10 mV increments in 3 s intervals) (Figure 2C and D). The peak *I*-*V* curves are shown in Figure 2E. The peak *I*_Ba_ density was significantly increased in colonic neurons of TNBS treated rats compared with control (Figure 2F; -127.82 ± 20.82 pA/pF Vs −91.67 ± 19.02 pA/pF, n = 9, *P < 0.05).

**Figure 3:**
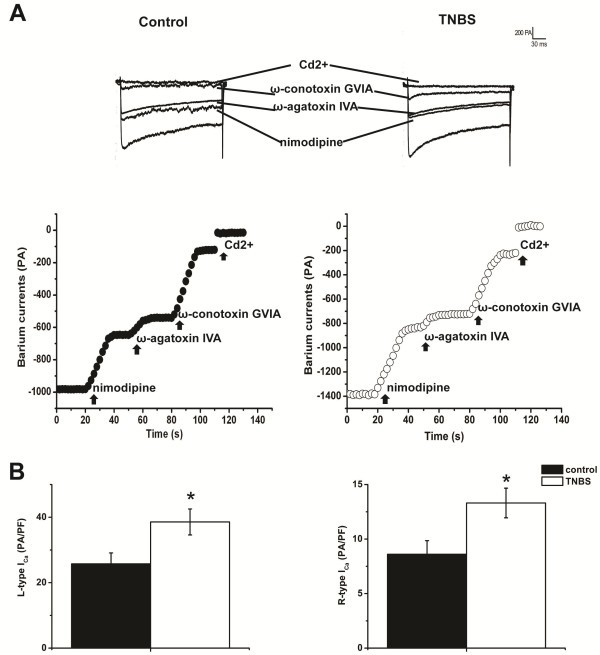
**Comparison of the L-type and R-type high voltage activated Ca**^**2+ **^**currents (HAV) of DiI**^**+ **^**DRG neurons from control and TNBS treated rats.** (**A**) Examples of the current traces (upper row) and the time course (lower row) with differrent calcium channel blockers on *I*Ba in colonic specific DRG neurons of control group (left, capacitance = 23.3 PF) and TNBS group (Right, capacitance = 24.8 PF). Currents were elicited by depolarizing neurons from −60 to 0 mV for 240 ms. (**B**) Changes of the current density of L-type and R-type current. The Bar graphs show that the density of L-type current (−38.56 ± 3.97 pA/pF Vs −25.75 ± 3.35 pA/pF, n = 9 for each group, * P < 0.05) and R-type current (−13.31 ± 1.36 pA/pF Vs −8.60 ± 1.25 pA/pF, n = 9 for each group, *P < 0.05) significantly increased in colonic specific DRG neurons of TNBS group compared with control group.

To distinguish different type of calcium currents (L-, N-, P/Q and R-type), the corresponding selective channel blockers nimodipine (5 μM, L-type), ω-conotoxin GVIA (1 μM, N-type), ω-agatoxin IVA(400 nM, P/Q-type) and Cd^2+^ (300 μM, R-type) were applied to the recording chamber. In this protocol, the cells were depolarized from −60 to 0 mV for 240 ms to elicit the high voltage activated *I*Ba. The representative current traces and time course are shown in Figure 3A. The fraction of L-type *I*Ba (−38.56 ± 3.97 pA/pF Vs −25.75 ± 3.35 pA/pF, n = 9, * P < 0.05) and R-type *I*Ba (−13.31 ± 1.36 pA/pF Vs −8.60 ± 1.25 pA/pF, n = 9 for each group, *P < 0.05) were significantly greater in colonic DRG neurons of TNBS treated rats than control rats (Figure 3B). There was no significant difference in the fraction of N- and P/Q-type calcium currents reduced by ω-conotoxin GVIA and ω-agatoxin IVA.

### The effect of intrathecal injection of nimodipine and SNX-482

**Figure 4:**
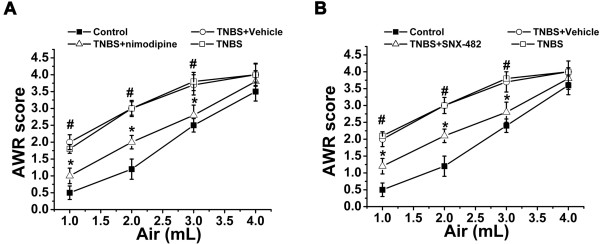
**Summary data of the mean AWR to CRD following TNBS in rats treated with vehicle or nimodipine and vehicle or SNX-482. A**. The AWR score significantly increased at distension volume at 1.0 2.0 and 3.0 ml 4 days following intracolonic TNBS compared with control rats (n = 9, # p < 0.05) indicating the development of visceral hyperalgesia. There was a significantly decrease in the AWR in the TNBS treated rats that received nimodipine (n = 9, * P < 0.05) and there was no difference in the AWR in the rats that received vehicle. **B**. The AWR score significantly increased at distension volume at 1.0 2.0 and 3.0 ml 4 days following intracolonic TNBS compared with control rats (n = 9, # p < 0.05). There was a significantly decrease in the AWR in the TNBS treated rats that received SNX-482 (n = 9, * P < 0.05) and there was no difference in the AWR in the rats that received vehicle.

Compared with control rats, the TNBS treated rats had significantly higher response to colonic distension (at 1, 2 and 3 ml distention volumn), indicating that TNBS treated rats were more sensitive to CRD which was called visceral hypersensitivtiy (Figure 4A, B, # P < 0.05). To investigate whether elevated L-type and R-type calcium current in colonic DRG nerons plays a causal role in TNBS induced inflammatory visceral allodynia, we treated the TNBS clystered rats with L-type (nimodipine) and R-type (SNX-482) selective channel blockers by intrathecal injection. The result showed that the intrathecal injection both nimodipine and SNX-482 significantly reduced visceromotor response to CRD at 1, 2 and 3 ml distention volumn (Figure 4A, B, * P < 0.05), while the vehicle treatments had no effect. The visceral mechanosensitivity of control rats did not change following intrathecal channel blockers.

## Discussion

In this study, we perform a microarray screening for genes with different expression in rat models of inflammatory visceral pain and demonstrate that Cav1.2 and Cav2.3 participates in the visceral hyperalgesia induced by TNBS-colitis. The result revealed that the level of Cav1.2 and Cav2.3 protein in L6-S2 DRG were significantly up-regulated following colitis and L-type and R-type calcium currents significantly increased in colonic specific DRG neurons, while visceral sensitivity to mechanical stimuli also increased significantly. Intrathecal administration of the specifically inhibits nimodipine and SNX482 reduced the colonic hyperalgesia to balloon distention.

Genes regulated in the L6-S2 DRG with TNBS treated may contribute to a variety of biologic processes such as inflammatory visceral pain signaling. In this study, we found that a large number of ion channels strongly regulated in DRG after TNBS treated such as VGCC (Cav1.2, Cav2.3), inositol 1,4,5-trisphosphate receptor 2, ryanodine receptors 1(RyR1), P2X7, P2Y and so on. Some studies have reported about the role of these channel in pain sensation such as RyR1 [7] P2X7 [8-13] and P2Y1 [14]. But these channels haven’t been studied in visceral sensation. In addition, P2X7 and P2Y1 receptors involved in the neuronal soma-satellite cell interaction [13,15]. Maybe the up-regulation of P2X7 and P2Y1 can improve the neuron–glia communication. We also found that a large number of neurotransmitter and receptor strongly regulated in DRG after TNBS treated such as tumor necrosis factor (TNF, member 2 and 4), PGER(EP2), 5-HTR 1A, 5-HTR 2A and FGFR3, which upregulated in DRG of TNBS treated rat. Recently, several studies provided evidence that TNF-α may act on primary afferent neurons and induces hypersensitivity [12,16,17]. Whether the up-regulation of EP2 in L6-S2 DRGs of TNBS treated rats, mediating the visceral hypersensitivity, needed further study. The previous studies have suggested that 5-HT1A, 5-HT2A, 5-HT2C, 5-HT3 and 5-HT4 receptor subtypes in the DRG play important roles in facilitating peripheral nociceptive transmission [18-20]. Besides the ion channels and receptors, signal transduction molecules were also significantly changed. These changes could be associated with the rearrangement of pain sensory pathways that take place during the development of visceral inflammatory pain. All these changed genes maybe establish a positive feedback loop between neuron-neuron and neuron-satellite cell, thus contributing to exaggerated responses under pathological conditions which still need further experimental research proof.

In our study, we found that both the expression and the function of L- and R- type VGCC were upregulated in colonic DRG neurons of TNBS treated rats compare with control group. Kania has reported L-type VGCC blockers (nifedipine, diltiazem verapamil) can be useful in controlling acute visceral pain in the model of visceral pain or hypersensitivity of sheeps, which suggests that L-type VGCC play a crucial role in the modulation of acute visceral hyperalgesia [21-23]. They only use the pharmacology method in the behavioral test lacking the studies of molecular mechanism. In addition to visceral pain, neuronal L-type VGCCs Cav1.2 and Cav1.3 have reported to be up- or down regulated in the model of neuropathic pain in rats [24,25]. R-type Ca^2+^ channels located at primary synapses [26] and can contribute to neurotransmitter release [27]. R-type VGCCs are associated with nociceptive processing in various pain conditions. Mathews reported that spinal SNX-482, an R-type VGCC blocker, inhibited noxious C-fiber and Aδ-fiber-mediated dorsal horn neuronal responses in conditions of neuropathy, not in sham operated rats and that non-noxious Aδ-mediated responses did not affect by SNX-482 [28]. In another study, mice lacking the α1E Ca^2+^ channel subunit exhibited normal pain behavior against acute mechanical, thermal, and chemical stimuli; however, they showed reduced responses to somatic inflammatory pain [29]. So far, there has been no definitive study that shows that the role of R-type VGCC in visceral sensation. Our results revealed that upregulated Cav1.2 (L-type) and Cav2.3 (R-type) maybe involved in the inflammatory visceral hypersensitivity induced by TNBS treated.

The VGCCs manage and adjust neurotransmitter release and Ca^2+^-dependent depolarization that contribute to the characteristic firing patterns of most neurons, including those in pain pathways. As far, the role of VGCC in pain was not consensus. Because a change in [Ca^2+^]_i_ can affect neuronal properties via changes in ion channel activity [30,31], enzymatic activity [32] and/or gene expression [33], the physiological impact of the inflammation induced changes in evoked increases in [Ca^2+^]_i_ will depend on where in the neuron they are manifest. On the other hand, an increase in [Ca^2+^]_i_ close to functional Ca^2+^ dependent K^+^ channels should increase K^+^ channel activity resulting in a decrease in afferent excitability and the transmission of nociceptive information, while a change in the dynamics of Ca^2+^ signaling in the cell body may result in changes in gene expression that are either pro- or anti-nociceptive [34]. Thus, the colon inflammation-induced changes in the regulation of [Ca^2+^]_i_ may contribute to the visceral inflammatory hyperalgesia or serve as a feedback inhibitory mechanism functioning as a “break” to a host of pro-nociceptive inflammatory processes. We further detected the visceral pain after intrathecal injection of specific calcium channel inhibitors. Nimodipine specifically inhibits Ca_v_1.2 α1C and to a lesser extent T-type VGCC in central and peripheral neurons. But the concentration for T-type Ca^2+^ channel inhibition is two orders of magnitude higher than for L-type Ca^2+^ channel [35]. The SNX-482 is specific inhibitor for Cav2.3. Cav1 (L-type) can be partially inhibited by saturating concentrations of SNX-482 [36]. SNX-482 is a large peptide and the concentration of SNX-482 applied was 5 μg/Kg. The concentration in the tissue were limited and this concentration can’t inhibit the Cav1 [28]. As the intrathecal injection of nimodipine or SNX-482 significantly reduced the visceral inflammatory hyperalgesia in this study, we supposed that Cav1.2 and Cav2.3 may contribute to the visceral inflammatory hypersensitivity. However, additional experiments will be needed to study the direct consequence of the increase of [Ca^2+^]_i_ on function of the sensory neurons.

In summary, our data revealed that there are many genes marked changed in L6-S2 DRGs of visceral inflammatory model, which encompass a large number of distinct family members including neuropeptides, receptors, ion channels, signal transduction molecules and others. Among these changed genes, the up-regulation of Cav1.2 and Cav2.3 contributed to visceral inflammatory hyperalgesia, which maybe the potential therapeutic targets for visceral pain.

## Materials and methods

### Experimental colitis

Experiments were performed on male Sprague–Dawley (SD, 200 g-250 g) rats. All animal procedures used in this study were approved by Medicine Animal Care and Use Committee of Shanghai Jiaotong University School and were in accordance with the guidelines of the International Association for the Study of Pain. To induce inflammation in the distal colon, fasted rats were anaesthetized and TNBS (0.6 ml of 30 mg/ml TNBS in 50%Ethanol) was instilled into the lumen of the colon through a syringe-attached polyethylene catheter via the rectum 6 cm proximal to the anus. Animals that received an equal volume of 50% EtOH enema served as controls throughout these studies. All colonic instillations were performed under isoflurane (2%) anaesthesia.

### Agilent DNA microarray analysis

After the 4-day recovery period, four rats from each group were terminally anesthetized with CO_2_, and then the L6-S2 DRGs was rapidly removed and stored at −80°C. Total RNA of DRG (L6-S2) from each group was extracted using Trizol (Invitrogen Inc., Carlsbad, CA, USA) and purified with the RNeasy Mini Kit (Qiagen, Hilden, Germany). The cDNA used for microarray analysis was prepared by the SuperAmpTM PCR based amplification method performed according to Miltenyi Biotec’s undisclosed protocol. Probe labelling was performed according to Miltenyi Biotec’s undisclosed protocol. The cDNA was denatured for 4 min at 70°C and cooled down to room temperature. Afterwards, 11 μL 10× control target solution, 11 μL 10× blocking agent solution (Agilent), and 55 μl 2× Gene Expression hybridization buffer (Agilent) was added. The entire volume of the reaction was subsequently hybridized overnight (17 h, 65°C) on Agilent Whole Rat Genome Oligo Microarrays (4 × 44 K format) using Agilent’s recommended hybridization chamber and oven. The microarrays were washed once with 6× SSPE buffer containing 0.005% Nlauroylsarcosine for 1 min, followed by a second wash with 0.06× SSPE containing 0.005% Nlauroylsarcosine for 1 min, and a final wash step with acetonitrile for 30 s. The fluorescence signals of the hybridized Agilent Microarrays were detected using Agilent’s Microarray Scanner System (Agilent).

Data were imported into Genedata Expressionist Analyst (Genedata AG, Basel, Switzerland), and the Cyanine 3 fluorescence intensities normalized using lowest weighted linear regression (LOWESS). Normalized data in tab delineated text format were uploaded into NIA array analysis (http://lgsun.grc.nia.nih.gov/ANOVA); this allowed a pairwise comparison of gene signal intensity (expression) with fold difference of two or greater, as determined by single factor analysis of variance with multiple hypothesis correction by the false discovery rate. The fold change was calculated for each sample to represent a ratio of expression between TNBS treated and control samples.

### Validation of gene transcription by real-time PCR

RT- PCR was carried out on the same set of samples that were analyzed by the microarray approach. Three μg total RNA of each tested sample was used for reverse transcription. PCR primers were as follows:

Cav1.2 forward: TCAAAGGCTACCTGGACTGGAT

reverse: CCATGCCCTCGTCCTCATT

Cav2.3 forward: GCACTACATCTCTGAGCCCTATCTG

reverse: TCTCCTCCTCGCCACAGTCT

β-actin forward: TCTGTGTGGATTGGTGGCTCT,

and reverse: AGAAGCATTTGCGGTGCAC.

Quantitative PCR was performed in 25 μL with the ABI Power SYBR Green PCR Master Mix (ABI, USA) on the 7300 Sequence Detection System (ABI, USA). Thermal cycling was initiated with incubation at 50°C for 2 min and 95°C for 10 min. After this initial step, 40 cycles of PCR were performed. Each PCR cycle consisted of heating at 95°C for 15 s for melting and 60°C for 1 min for annealing and extension. All RT-PCRs were performed in triplicate, and the results were normalized with the *Ct* values of β-actin.

### Western blot

The western blot for protein expression of DRGs was done as previous reported [37]. Animals (5 rats of each group) were terminally anesthetized with CO2, the L6-S2 DRGs-containing colon afferent rapidly removed, and stored at −80°C. Protein extracts from pooled DRG (L6-S2) were prepared in SDS buffer: 50 mM Tris–HCl, 133 mM NaCl, 2%SDS, 1 mM DTT, 1 mM PMSF, 1:100 dilution of protease inhibitor cocktail (sigma), pH = 8. Twenty-five micrograms (25 μg) of protein were loaded onto 9% SDS-PAGE, and electrophoretically transferred to PVDF membrane (Bio-Rad, Hercules, CA) at 100 V for 2 h. The membranes were blotted with antibodies against Cav1.2 and Cav2.3 (sigma, dilution 1:200), followed by incubation with horseradish peroxidase (HRP)-conjugated secondary antibody (Dako Cytomation, Denmark). Bands were visualized using ECL (Amersham) kit and appropriate exposure to Kodak X-ray film. Films were scanned and band intensities measured using Gel-pro Analyzer 4.0 software (Media Cybernetics). Cav1.2 and Cav2.3 protein expression were normalized to β-actin.

### Isolation and identification of distal colon projecting DRG neurons

Colon specific DRG neurons were labelled by injection of 1,1^′^-dioleyl-3,3,3^′^,3-tetramethylindocarbocyanine methanesulfonate (DiI, Invitrogen) into the colon wall, which has been described previously report [37]. After 1 wk of DiI labeling, TNBS colitis was induced as previous. 4 days later, colonic DRG neurons from both groups were used for recording voltage-gated calcium current. Isolation of DRG neurons from these adult SD rats has been described previously report [37].

### Electrophysiological recordings

The whole cell patch clamp recording of DiI^+^ has been described previously report [37]. Patch electrodes with a resistance of 2–4 MΩ were pulled from borosilicate glass capillaries (ID 0.86 mm, OD 1.5 mm, Length 10 cm, Sutter Instruments) using a micropipette puller (P-97 Sutter Instruments, Novato, CA). Neurons were patched in the whole cell configuration and recorded using an EPC-10 amplifier (HEKA Instruments, Lambrecht, Germany). Seals (1–10 GΩ) between the electrode and the cell were established. After whole cell configuration was established, the cell membrane capacitance and series resistance were electronically compensated. Recordings were only made when access resistance fell to <15 MΩ. Up to 80% of the series resistance was compensated electronically. Leak currents were subtracted using the on-line P/4 protocol. Barium currents (*I*Ba) flowing through calcium channels were recorded using extracellular solution consisting of (in mM): 140 TEA-Cl, 2 MgCl2, 3 BaCl2, 10 glucose, 10 HEPES (pH 7.4 adjusted with TEA-OH, osmolarity 320). The pipette solution contained (in mM): 120 CsCl, 1 MgCl2, 10 HEPES, 10 EGTA, 4 Mg-ATP and 0.3 Na-GTP (pH 7.2 adjusted with CsOH, osmolarity 300 mOsm). To minimize the “run-down” associated with the whole-cell recording, GTP and ATP were included in the pipette solution. The total *I*Ba was elicited by a 300 ms voltage step from −80 to 50 mV with 10 mV increments in 3 s intervals (holding potential, −100 mV). The high voltage actived *I*Ba was elicited by a 240 ms voltage step from −60 to 0 mV (holding potential, −60 mV). ω-conotoxin GVIA and ω-agatoxin IVA were dissolved in distilled water at 1000 times the final concentration and kept frozen in aliquots. Nimodipine was prepared as a stock solution dissolved in DMSO. To distinguish the L-, N-, and P/Q-type calcium currents in DiI^+^ DRG neurons, the corresponding selective channel blockers nimodipine (5 μM, L-type), ω-conotoxin GVIA (1 μM, N-type), ω-agatoxin IVA (400 nM, P/Q-type) and Cd^2+^ (300 μM, R-type) were applied to the same recording neurons. The stock solutions were diluted in extracellular solution just before use and held in a series of independent syringes connected to corresponding fused silica columns (ID 200 μm). Each drug solution was delivered to the recording chamber by gravity flow, and rapid solution exchange was achieved by controlling the corresponding valve switch (World Precision Instruments). All drugs and chemicals were all purchased from Sigma.

### Intrathecal catheters

Chronic intrathecal catheters were implanted three days before TNBS induced colitis. Following anaesthetized with 1% pentobarbital sodium 100 mg/kg intraperitoneally. The L3 and L4 vertebrae were exposed. A 32-gauge polyimide catheter was then threaded into the subarachnoid space and passed in a caudal direction for approximately 3 cm. The external end of the catheter was connected to a length of PE-10 polyethylene tubing which was tunnelled and exteriorised over the forehead. Animals were then caged individually. Any animal developing motor impairment following catheter placement was excluded from the study. All the drugs were given intrathecally in a total volume of 10 μL. The channel blockers were injected twice daily for 4 d, starting from the TNBS treated day. The nimodipine was dissolved in DMSO and SNX-482 was dissolved in sterile saline. Nimodipine (20 μg/kg) and SNX-482(5 μg/kg) were administered as a bolus via the intrathecal catheter in volumes of 10 μl. The catheter was flushed afterwards with 10 μl saline. The same amount of vehicle (DMSO or 0.9%NaCl) was injected into some TNBS treated rats as vehicle control. Behavioral testing was performed after the last injection on the fourth day of TNBS treatment as described below.

### Behavioral testing for nocifensive response

We used a grading system based on the abdominal withdrawal reflex (AWR) in response to colorectal distention (CRD) to evacuate the visceral sensitivity. Briefly, under sedation with aether, a flexible latex balloon (5 cm) attached to the perforated end of an arterial embolectomy probe (LeMaitre Embolectomy Catheter 6F; LeMaitre Vascular) was inserted 6–8 cm into the descending colon and rectum via the anus and held in place by taping the tubing to the tail. CRD was performed by rapidly inflating the balloon to a constant volumn. The balloon was inflated to 1, 2, 3 and 4 ml, for 20 s followed by 2 min rest. Behavioral response to CRD was measured by visual observation of the AWR by an observer blind to the experimental conditions, and AWR was scored as: 1 (normal behavior), 2 (contraction of abdominal muscles), 3 (lifting of abdominal wall), or 4 (body arching and lifting of pelvic structures).

### Statistical analysis

Data were statistically analyzed by analysis of variance (ANOVA) and sequential differences between means were tested by Tukey contrast analysis at P < 0.05.

## Abbreviations

DiI: 1,1^′^-dioleyl-3,3,3^′^,3-tetramethylindocarbocyanine methanesulfonate; TNBS: 2,4,6-trinitrobenzenesulfonic acid; AWR: Abdominal withdrawal reflex; CRD: Colorectal distention; DRG: Dorsal root ganglia; [Ca2+]i: Intracellular calcium concentration; PGE2: Prostaglandin E2; Ryr1: Ryanodine receptor 1; TNF-α: Tumor necrosis factor –α; VGCC: Voltage gated calcium channel

## Competing interest

The authors declare that they have no competing interests.

## Authors’ contributions

AQ carried out the path clamp studies; performed the statistical analysis and drafted the manuscript. DS and DT carried out rat behavior studies and molecular genetic studies. YL and XL were responsible for technical support; WY carried out western blot studies and was responsible for critical revision of the manuscript for important intellectual content. YY conceived of the study, and participated in its design and coordination and helped to draft the manuscript. All authors read and approved the final manuscript.
